# Analysis of binding properties and specificity through identification of the interface forming residues (IFR) for serine proteases *in silico *docked to different inhibitors

**DOI:** 10.1186/1472-6807-10-36

**Published:** 2010-10-20

**Authors:** Cristina Ribeiro, Roberto C Togawa, Izabella AP Neshich, Ivan Mazoni, Adauto L Mancini, Raquel C de Melo Minardi, Carlos H da Silveira, José G Jardine, Marcelo M Santoro, Goran Neshich

**Affiliations:** 1 Department of Biochemistry and Immunology, Institute of Biological Sciences, Federal University of Minas Gerais, Belo Horizonte, MG, Brazil; 2 Embrapa Genetic Resources and Biotechnology, Brasilia, DF, Brazil; 3 Embrapa Information Technologies, Campinas, SP, Brazil; 4 Department of Mathematics and Computer Science - Federal University of Itajubá, Itajubá, MG, Brazil; 5 Department of Computer Science, Institute of Exact Sciences, Federal University of Minas Gerais, Belo Horizonte, Brazil

## Abstract

**Background:**

Enzymes belonging to the same super family of proteins in general operate on variety of substrates and are inhibited by wide selection of inhibitors. In this work our main objective was to expand the scope of studies that consider only the catalytic and binding pocket amino acids while analyzing enzyme specificity and instead, include a wider category which we have named the Interface Forming Residues (IFR). We were motivated to identify those amino acids with decreased accessibility to solvent after docking of different types of inhibitors to sub classes of serine proteases and then create a table (matrix) of all amino acid positions at the interface as well as their respective occupancies. Our goal is to establish a platform for analysis of the relationship between IFR characteristics and binding properties/specificity for bi-molecular complexes.

**Results:**

We propose a novel method for describing binding properties and delineating serine proteases specificity by compiling an exhaustive table of interface forming residues (IFR) for serine proteases and their inhibitors. Currently, the Protein Data Bank (PDB) does not contain all the data that our analysis would require. Therefore, an *in silico *approach was designed for building corresponding complexes

The IFRs are obtained by "rigid body docking" among 70 structurally aligned, sequence wise non-redundant, serine protease structures with 3 inhibitors: bovine pancreatic trypsin inhibitor (BPTI), ecotine and ovomucoid third domain inhibitor. The table (matrix) of all amino acid positions at the interface and their respective occupancy is created. We also developed a new computational protocol for predicting IFRs for those complexes which were not deciphered experimentally so far, achieving accuracy of at least 0.97.

**Conclusions:**

The serine proteases interfaces prefer polar (including glycine) residues (with some exceptions). Charged residues were found to be uniquely prevalent at the interfaces between the "miscellaneous-virus" subfamily and the three inhibitors. This prompts speculation about how important this difference in IFR characteristics is for maintaining virulence of those organisms.

Our work here provides a unique tool for both structure/function relationship analysis as well as a compilation of indicators detailing how the specificity of various serine proteases may have been achieved and/or could be altered. It also indicates that the interface forming residues which also determine specificity of serine protease subfamily can not be presented in a canonical way but rather as a matrix of alternative populations of amino acids occupying variety of IFR positions.

## Background

Serine proteases play an important role in processes such as blood clotting, digestion and in some pathways of cell development [1]. Serine proteases can hydrolyze either peptide bonds or esters. Proteases digest proteins by hydrolyzing the peptide bonds which are responsible for keeping amino acids together [2,3]. The cleavage specificity of elastase, trypsin, chymotrypsin and other serine proteases depends on the volume/size, form/shape, and polarity/charge/hydrophobicity of the specific part of a protein surface where a substrate will be docking - the specificity pocket [4,5]. There are three amino acid residues responsible for the enzymatic activity that are present in all serine proteases, which are denominated as the catalytic triad: His 57, Asp 102 and Ser 195 (chymotrypsin numbering system is used throughout - see [6]). Interestingly, out of those three amino acids, only Asp 102 does not make part of the interface (the definition of which is based on decreased solvent accessible area upon substrate/inhibitor binding). This is due to the fact that Asp 102 is already not accessible to solvent in isolated enzyme, because this amino acid is located at the very bottom of the active site cleft where solvent molecules (water) can not access it. The role of the first two amino acids (His 57 and Asp 102) during trypsin catalysis, for example, is to function as a proton shuttle. Trypsin cleaves peptides after Lys and Arg residues with the co-participation of Asp 189, which interacts with the positive charge on peptide [7,8]. Chymotrypsin, on the other hand, cleaves proteins after aromatic (and also large hydrophobic) residues [9]. To achieve such specificity, in chymotrypsin, one can easily identify the existence of the hydrophobic pocket, normally shielded by Met 192. Thrombin is a protease which cleaves peptides with more specificity than trypsin: it requires Arg on "P1" position [10,11]. Our main objective in this work is to expand the scope of studies that analyze enzyme specificity by including into observations not only the catalytic triad and binding pocket but also a wider category of amino acids which we have named the Interface Forming Residues (IFR) [12-15]. Namely, only a part of the molecular surface is shielded from solvent upon formation of a bi-molecular complex. The residues having a lower accessibility to solvent upon complex formation have an important role in the process of docking and also in defining specificity [15,16]. Therefore, we were motivated to calculate which amino acids are shielded from solvent in the bi-molecular complexes involving serine proteases and different types of inhibitors and then create a table (matrix) of all amino acid positions at the interface and their respective occupancies. By mapping those amino acids as a specific IFR, we are now able to analyze characteristics of each position; and by doing so, we also can make position-specific alignment among different subfamilies of serine proteases. The key step we needed to solve during our procedural approach was to find a sufficient number of PDB [17] structures containing complexes of serine proteases with respective inhibitors. It became clear that we would need to either produce those by some novel method or abandon our work because there were not enough samples available in the PDB. The solution to this challenge is presented in details in materials and methods.

## Results

### Interface Forming Residue (IFR) tables

In order to provide a sufficient volume of data for analysis of the interfaces around active sites of serine proteases (even though there is no enough structural information available currently on complexes formed by one particular protease with any specific substrate and/or inhibitor) we needed to employ the *in silico *approach for building corresponding complexes. The key feature of our work is mapping the IFR 3D profile into a 2D matrix--from a known enzyme-inhibitor structure to those with no known structure for such complex. Mapping is done after structurally aligning all serine proteases with non-redundant sequences.

**Figure 1:**
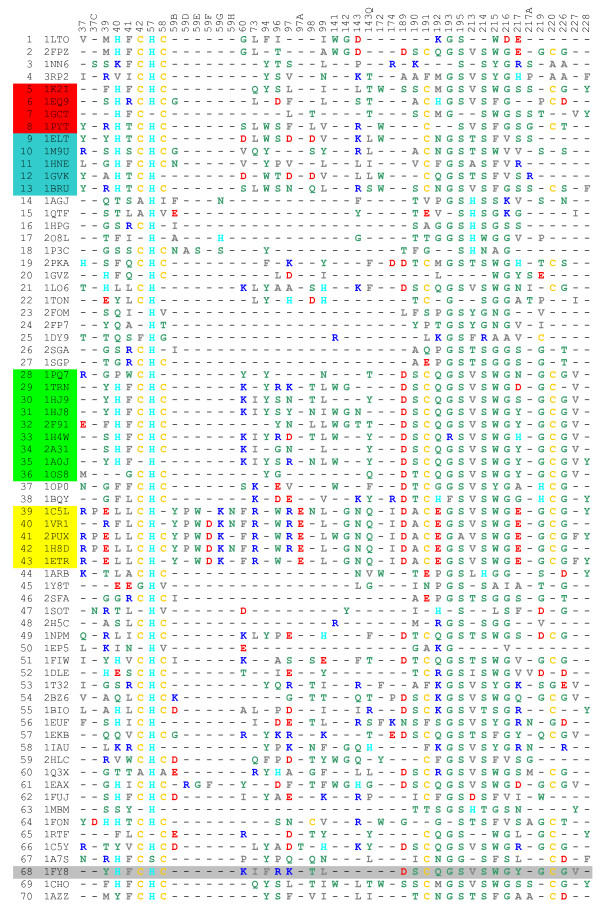
**The IFR positions and their occupancy by amino acids shown for 70 serine proteases bound to BPTI**. The rows show amino acids occupying IFR positions for specific serine protease (the PDB ID given in the second column). The top row shows the sequence numbers in structurally aligned serine proteases (only IFRs are listed) for all enzymes in complex with the BPTI. The complexes were created by the procedure we named "hard docking", except for the case of 1fy8.pdb for which we have experimental data (row marked with light gray background). The color code for amino acids is as follows: Residues: **AVLIMFP **are colored grey [containing: small hydrophobic residues (**A **and **P**) and large hydrophobic residues]; Residues: **STYNQWG **are colored green [containing: polar residues]; Residues: **D **and **E **are colored red [containing: negatively charged residues]; Residues: **R **and **K **are colored blue [containing: positively charged residues]; Residue: **H **is colored cyan [containing: positively charged residue which is neutral at neutral pH]; Residue: **C **is colored yellow [containing: disulphide bridge forming residue]. {See text for details about amino acid classification} The rows are occupied by following serine protease sub-families: Tryptase(1-2), Chymase (3-4), Chymotrypsin (5-8) {red background}, Elastase (9-13){blue background}, Exfoliative toxin (14-15), Glutamyl endopeptidase (16-18), Kallikrein (19-22), NS3 protease (23-25), Streptogrisin (26-27), Trypsin (28-36){green background}, Venom (37-38), Thrombin (39-43){yellow background}, Miscellaneous-Prokaryotes (44-48), Miscellaneous-Virus (49-50), Miscellaneous (51-67), Native Complex (68-70) in respective row order.

After selection of 67 serine protease structural files (in a way described in materials and methods), named here as a secondary datamart, and three prototype complexes, containing inhibitor/serine protease complexes, we ended up with total of 70 structures with non redundant enzyme sequences. The list of all corresponding PDB IDs for serine proteases used in this work is presented in the Table 1. All selected 70 serine proteases were then structurally aligned. Upon rigid body docking of the three different inhibitors to 67 serine proteases from the secondary datamart, we constructed complexes which, counting also the three prototype ones, totalled to three sets of 70 two-chain macromolecules. The interfaces for those three sets were calculated using SurfV program [18] and for each *in silico *generated complex, we mapped all of the sequence positions that belonged to IFR ensemble into the table shown at Figure 1.

**Table 1:** List of all PDB files containing serine protease structures used for structural alignment

01-1LTO	02-2FPZ	03-1NN6	04-3RP2	05-1K2I
06-1EQ9	07-1GCT	08-1PYT	09-1ELT	10-1M9U

11-1HNE	12-1GVK	13-1BRU	14-1AGJ	15-1QTF

16-1HPG	17-2O8L	18-1P3C	19-2PKA	20-1GVZ

21-1LO6	22-1TON	23-2FOM	24-2FP7	25-1DY9

26-2SGA	27-1SGP	28-1PQ7	29-1TRN	30-1HJ9

31-1HJ8	32-2F91	33-1H4W	34-2A31	35-1A0J

36-1OS8	37-1OP0	38-1BQY	39-1C5L	40-1VR1

41-2PUX	42-1H8D	43-1ETR	44-1ARB	45-1Y8T

46-2SFA	47-1SOT	48-2H5C	49-1NPM	50-1EP5

51-1FIW	52-1DLE	53-1T32	54-2BZ6	55-1BIO

56-1EUF	57-1EKB	58-1IAU	59-2HLC	60-1Q3X

61-1EAX	62-1FUJ	63-1MBM	64-1FON	65-1RTF

66-1C5Y	67-1A7S	**68**-1FY8	**69**-1AZZ	**70**-1CHO

The last three PDB IDs (underlined) contain a description of the native complexes of: anionic trypsinogen with **BPTI**, collagenase with **Ecotin **and alpha-chymotrypsin with **Ovomucoid**, respectively.

Analysis of the tabulated IFR data (as presented in the Figure 1) clearly indicates subfamily differences with respect to characteristics, position and frequency of occupancy of interface residues. The data compiled here can be used as an additional tool for characterizing function and specificity. This is particularly relevant for planning structure/sequence changes intended to alter protein activity, e.g., from trypsin to chymotrypsin type activity. More generally, one subfamily type of serine protease can be adjusted to behave like another if corresponding changes are made in an initial protease sequence. Attempts have been made to alter protein activity [4,19], but they have focused on a single or, at most, 5 to10 residues. Our data, shown on Figure 1, indicate that such alterations can be achieved by eliminating key differences in IFR occupancy.

**Figure 2:**
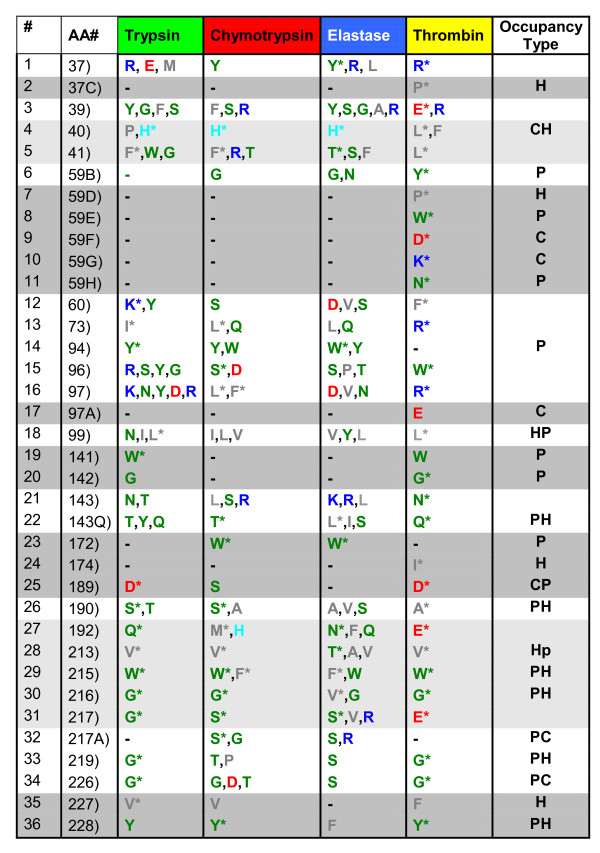
**An extract from all IFR positions showing the most restrictive ones, identified for four serine proteases subfamilies, bound to BPTI**. The positions shown are among the most restrictive ones in terms of the type of residues occupying them in 4 sub families of serine proteases: trypsins; chymotrypsins; elastases and thrombins, respectively. Those residue types that are present in at least 50% or more of analyzed proteases within a single subfamily, at a given IFR position, are indicated by a '*' sign. The colors of residues correspond to STING AA color coding {see text for details as well as the legend for the Table 1}: green are **P**olar, gray are **H**ydrophobic, red are negatively **C**harged, blue (and cyan for His) are positively **C**harged residues. The gray background color is used to indicate those IFR positions which are populated, within a sub family, by a single residue type or are absent from the IFR ensemble (represented by "-"). Those IFR positions occupied in each of four sub families by one predominant class of amino acid (indicated by "*") are labeled in light gray.

**Figure 3:**
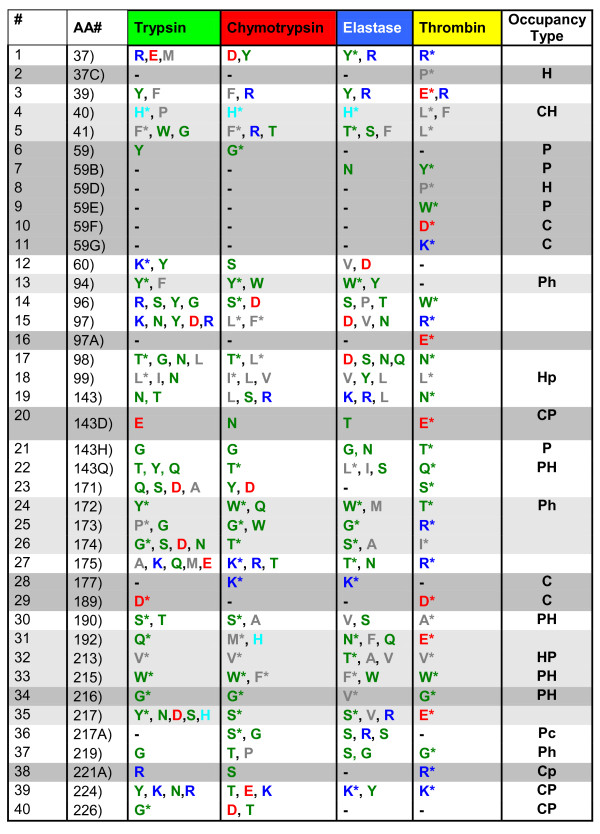
**An extract from all IFR positions, identified for four serine proteases subfamilies, bound to Ecotine**. Shown positions are designated in a same form as in the Figure 2 above.

In case of trypsin, chymotrypsin, elastase and thrombin, each bound to BPTI, differences are present at 36 IFR positions (presented in the table shown at Figure 2). In the table shown at Figure 3, for instance, sequence position 40 is occupied by His (in chymotrypsin and elastase exclusively, and in trypsin mainly) or hydrophobic residues (in thrombins exclusively). Position 172 (across all 4 subfamilies) is occupied by mostly polar residues (with some hydrophobic residues present in elastase case). Positions 94, 215 and 216 are occupied by mostly polar residues in at least 3 subfamilies.

In general, positions were designated as "**Ph**" if they were occupied by mostly (present in at least 50% of complexes) **P**olar and occasionally some **h**ydrophobic residues. Similarly, IFR positions occupied by only **C**harged residues were marked as "**C**", those with only **P**olar residues as "**P**" and those occupied by only **H**ydrophobic residues as "**H**". A combination of letters would mean presence of residues belonging to multiple/distinct amino acid classes. Capital letters are used to designate a predominant residue presence (again, present in at least 50% of complexes analysed) while small letters designate an occasional presence.

There are less restrictive positions which can be occupied by different classes of amino acids in either one sub family (such as position 143 and 175) or across all subfamilies (positions 37, 60, 96, 97, 192 and 224). A specific analysis of the position 97, shows that it is less restrictive for trypsins (positively charged: K, R; polar: N, Y; negatively charged: D) and elastases (polar: N; negatively charged: D; hydrophobic: V) than for chymotrypsins (hydrophobic: L, F) and thrombins (positively charged: R).

**Figure 4:**
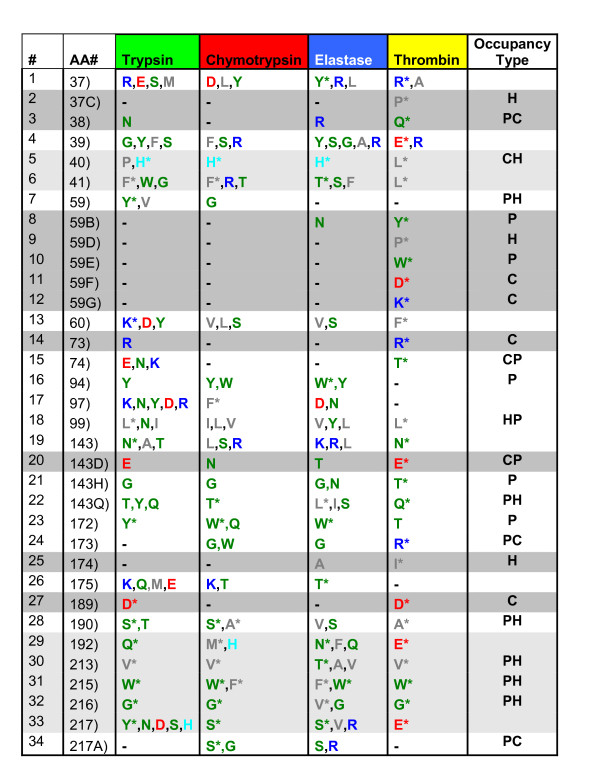
**An extract from all IFR positions, identified for four serine protease subfamilies bound to Ovomucoid third domain**. Shown positions are designated in a same form as in the Figure 2 and 3 above.

In order to additionally facilitate analysis of the data at Figures 2, 3 and 4, we have labelled with the gray background color those IFR positions which are populated, within a subfamily, by a single residue type or are absent from the IFR ensemble (represented by "-"). It is important to realize that the presence of one type of amino acid per subfamily is treated here in a same fashion as the absence of that particular position from the IFR ensemble because we understand that the event we call "no-show" for particular position is equally information dense as the information on type of occupancy for particular IFR position. Those IFR positions occupied in each one of four sub families by one predominant type of amino acid, present in at least 50% of serine proteases showing this particular IFR, marked here with the "*", are labelled in light gray. Therefore, the analysis of Figures 2, 3 and 4 is aided by four distinct labels: 1) residue "aminochromography", 2) dark gray background, 3) light grey background and 4) "occupancy type". The fourth label is present in the last column and indicates the two predominant residue classes present in the preceding 4 columns (four serine protease subfamilies). Similar analysis was done for two other inhibitors: BPTI with 36 identified IFR positions at the enzyme side (Figure 2), and ovomucoid third domain with 34 identified IFR positions at the enzyme side (the table shown at Figure 4).

From the data presented in Figures 2 and 4 (restrictive IFR positions of complexes formed with BPTI and ovomucoid third domain inhibitors, respectively) IFR locations 94 and 172 are shown to contain only polar residues (no presence of hydrophobic residues was found in contrast to the same locations in the Figure 3). By comparing the data from the figures 2, 3 and 4, the complexes of serine proteases with ecotine inhibitor compile 13 restrictive (gray), 10 half-restrictive (light gray) and 17 non-restrictive IFR positions (total: 40 positions). In the case of the BPTI and ovomucoid inhibitors, respective data are: 14, 7 and 15 (36); 11, 7 and 16 (34). Those numbers indicate that ecotine binding to serine proteases involves 32.5% restrictive, 25% less restrictive and 42.5% non restrictive IFR positions while BPTI and ovomucoid show respectively: 39%, 19.5% and 41.5%; 32%, 20.5% and 47%. Consequently, these numbers show that the ovomucoid inhibitor binding to serine proteases is less specific than the binding of ecotine, which in turn is less specific than BPTI. The experimental Ki [M] values reported in the literature for BPTI against trypsin is 6 × 10^-14 ^while for the chymotrypsin it goes down to 9 × 10^-9 ^and for elastase to 3.5 × 10^-6 ^(ref: http://www.gbiosciences.com/ResearchProducts/Aprotinin.aspx). On the other side, turkey ovomucoid third domain was reported to interact with very similar association constant with eight different serine proteinases-bovine chymotrypsins A and B, porcine pancreatic elastase I, proteinase K, Streptomyces griseus proteinases A and B, and subtilisin-Carlsberg [20]. Pál et al. [21] reported the following Ki (M) values for ecotine against trypsin, chymotrypsin and elastase, respectively: 1 × 10^-12^, 4 × 10-^12 ^and 1.3 × 10^-9^.

### Amino acid composition at serine protease interfaces

**Figure 5:**
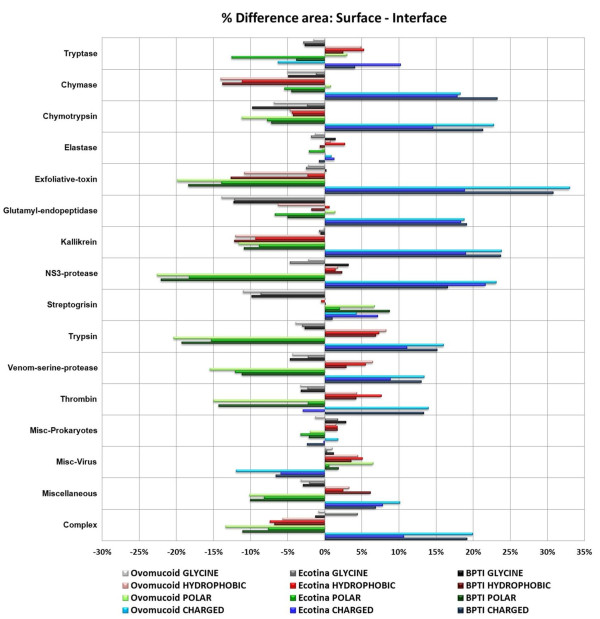
**Difference of residue class occupancy in total enzyme free surface vs. interface**. This Figure presents the difference in occupancy percentage of total enzyme free surface and the IFR area for all 70 serine proteases bound to the inhibitor Ecotine, BPTI and Ovomucoid third domain. The enzymes were classified in the following subfamilies: Tryptase (2), Chymase (2), Chymotrypsin (4), Elastase (5), Exfoliative Toxin (2), Glutamyl endopeptidase (3), Kallikrein (4), NS3 protease (3), Streptogrisin (2), Trypsin (9), Venom (2), Thrombin (5), Miscellaneous-Prokaryotes (5), Miscellaneous-Virus (2), some variety of serine proteases not belonging to any of above defined subfamilies (Miscellaneous, 17) and experimental complex (3). Average values of percent occupancy are presented for multi-member subfamilies. The numbers between the brackets show the number of enzymes in each subclass. Bars on the left side of the graph indicate that that particular residue class is more frequently found at the interface than at the surface. Bars on the right side of the graph indicate that the residues are more frequently found at the surface than on the interface.

Additionally, the difference between the polarity and hydrophobicity of the enzyme surface and interface was investigated for each *in silico *complex formed. The buried area and total enzyme free surface area (not including the interface) was compared with respect to the type of residues present. Again, the following residue classes were used: charged, hydrophobic and polar (with glycine considered separately, although being classified as a polar residue). The data presented in Figure 5 indicate that most interfaces, in all serine protease subfamilies bound to either BPTI, ecotine or ovomucoid third domain, have a general preference for polar residues (green and gray bars). Conversely, most of free enzyme surfaces (not including interface/buried area) show a preference for charged residues (blue bars) although some (7 out of 16) also do show a smaller (as compared to a preference for charged residues) preference for hydrophobic residues (red bars).

Some subfamilies do not follow these patterns. The interface of the streptogrisin subfamily shows a high presence of glycine residues for complexes with each of the three inhibitors (negative gray bars in Figure 5), while the rest of the surface shows preference for polar residues (green bars). Elastase shows the lowest differences among interface and surface preferences for 4 classes of amino acids defined in this work. Charged residues were found to be uniquely prevalent at the interfaces between the "miscellaneous-virus" subfamily and the three inhibitors. This prompts speculation about how important this difference in IFR characteristics is for maintaining virulence of those organisms. The upper part of the Figure 5 shows a strong presence of hydrophobic residues at the interfaces (together with polar residues including glycine), while the bottom part of the Figure 5 shows an absence (except for the 3 experimental complexes) of the hydrophobic residues at the interface (left portion of this graph). The bottom part of the Figure 5 shows an increase in the presence of hydrophobic residues at the free enzyme surface area. The surfaces (not including interface areas) have a prevalence of charged residues while the interface contains more polar (including glycine) residues (with some exceptions as described above). Thus, the IFR pocket of serine proteases is not formed by predominantly hydrophobic residues; it is a rather polar environment. Also, the interface contains much fewer (with one exception being "miscellaneous-virus") charged residues (as compared to the rest of the protease surface).

If our data are compared with the ones from Janin et. al. [22], one can observe that the serine proteases are endowed with a specific category of interfaces in terms of the contribution of the 20 amino acids types to that area: subfamilies vary in the contribution of hydrophobic residues and are almost constant in having a lower preference for the charged residues, as presented at the Table 2. These results point to the fact that although all four serine protease subfamilies do not show a great variability with respect to the percentage of the enzyme total surface area being buried upon *in silico *complex formation with the BPTI, ecotine and ovomucoid third domain (6.7%, 9.5% and 7.2% respectively, representing value of approximately 610 to 990 Å^2 ^of buried area), the percentage of the residue classes contributing to the interface varies significantly. The percentage that charged and polar residues contribute together to the total buried area in trypsin/BPTI is 86%, in chymotrypsin/BPTI is 65%, in elastase/BPTI is 75%, and for thrombin/BPTI is 84%. For all three inhibitors examined, thrombin and trypsin have the highest percentage of charged and polar residues contributing to buried area while elastase and chymotrypsin have the lowest. The details for the difference in occupancies are presented in Figure 5 and Table 2.

**Table 2:** The percent of the total enzyme buried area occupied by amino acid sub-classes

Inhibitor bound to serine proteases		[%] Hydrophobic residues in buried area	[%] Charged residues in buried area	[%] Polar residues at buried area (glycine %)	[%] (Charged + Polar) residues in buried area
BPTI	Trypsin (9)	14	10	76 (10)	86
	
	Chymotrypsin (4)	35	11	54 (8)	65
	
	Elastase (5)	25	21	54 (6)	75
	
	Thrombins (5)	16	37	47 (6)	84

Ecotine	Trypsin (9)	13	15	72 (10)	87
	
	Chymotrypsin (4)	30	18	52 (6)	70
	
	Elastase (5)	22	19	59 (9)	78
	
	Thrombins (5)	12	53	35 (6)	88

Ovomucoid Third domain	Trypsin (9)	12	10	78 (11)	88
	
	Chymotrypsin (4)	32	10	58 (9)	68
	
	Elastase (5)	24	20	56 (9)	86
	
	Thrombins (5)	15	37	48 (7)	85

The serine proteases are bound to BPTI, ecotine and ovomucoid third domain inhibitors. Areas occupied by the three classes of amino acids, with respect to the total buried enzyme area, are expressed as a percentage. The numbers between the brackets show the number of enzyme - inhibitor complexes included in this analysis for each subfamily. The column containing polar residue participation also contains (within brackets) the participation of glycine residues.

Those differences may be aligned with the corresponding function that each protease performs. However, it is important to realize the boundaries we should not cross when applying what we have learned here based on inhibitor-protease binary complexes to substrate-protease complexes. Nevertheless, we assume that inhibitors of serine proteases bind to the active site clefts of their target proteases in a manner that is thought to resemble the binding mode of substrates (Laskowski et al. [23]). Trypsin and thrombin handle the positive charges of a polypeptide by imposing the (complementary) charge/polarity of their own IFR surface. Chymotrypsin mitigates the spatial constraints imparted by large hydrophobic (and some polar) residues present in the substrate/inhibitor by accommodating glycines and hydrophobic residues into its own interface. Elastases, on the other hand, utilize hydrophobic amino acids among their IFRs to complement small, hydrophobic amino acids at the cleavage site of a polypeptide. The general trends for interfaces described above, when combined with the selectivity of IFR positions discussed earlier in this section, provide a more complete description of the specificity of serine protease subfamilies in both general and detailed way, although, as we expected from the beginning, there is no canonical code that we may convey from this analysis.

### Benchmarking the accuracy of the "hard docking" procedure for IFR identification

The "confusion matrix" presented at the Table 3 contains 5 categories of evaluators for IFR identification. The true positives (TP) are IFR residues present in both the native and the rigid body docked complexes. The true negatives (TN) are solvent exposed residues both in native and in *in silico *complexes. The false positives (FP) are residues present in the rigid body docked IFR table but absent in natural complexes and the false negatives (FN) are not buried in native complexes but appear in the IFR of the *in silico *complexes. The accuracy was calculated based on this formula: *ACC *= (*TP *+ *TN*)/(*P *+ *N*), where the P = (TP+FN) and N = (FP+TN). Table 3 shows these values for 1FY8 (control) and the sixteen complementary complexes. Overall, the accuracy (ACC) ranged between 0.97 and 0.98.

**Table 3:** Evaluation of the success rate (ACC) of predicting IFR ensemble

PDB ID	Subfamily	Organism	TP	TN	FP	FN	ACC
1FY8	Trypsin	*Bos taurus*	44	244	0	0	1

3TGI	Trypsin	*Bos taurus*	42	239	5	0	0.98

1TPA	Trypsin	*Bos taurus*	44	240	1	3	0.98

3BTK	Trypsin	*Bos taurus*	43	241	1	4	0.98

3TPI	Trypsin	*Bos taurus*	43	242	1	2	0.98

2PTC	Trypsin	*Bos taurus*	44	241	0	3	0.98

2TGP	Trypsin	*Bos taurus*	45	240	0	3	0.98

4TPI	Trypsin	*Bos taurus*	44	239	1	4	0.98

2TPI	Trypsin	*Bos taurus*	44	241	1	2	0.98

1BZX	Trypsin	*Bos taurus*	43	242	2	1	0.98

1BRB	Trypsin	*Rattus novergicus*	41	236	2	2	0.98

1FAK	Coagulation factor VIII	*Homo sapiens*	44	247	6	0	0.97

1CBW	Chymotrypsin	*Bos taurus*	44	238	5	1	0.97

1MTN	Chymotrypsin	*Bos taurus*	45	239	1	3	0.98

2KAI	Kallikrein	*Bos taurus*	57	224	0	4	0.98

1EAW	Matriptase MTSP1	*Homo sapiens*	42	241	4	1	0.98

The true positives (TP) are IFR residues present in both the native and the rigid body docked complexes. The true negatives (TN) are solvent exposed residues both in native and in in silico complexes. The false positives (FP) are present in the rigid body docked IFR but absent in natural complexes, and the false negatives (FN) are not buried in native complexes but appear in the IFR in generated in silico complexes.

### Evaluation of plausibility for *in silico *generated bi-molecular complexes

The presence of steric clashes involving enzyme and inhibitor residues was analyzed to evaluate our hard docking methodology. Steric clashes could yield unfavourable interactions within complexes that would lead to their decreased stability which might be translated to their faster de complexation *in vivo*.

**Figure 6:**
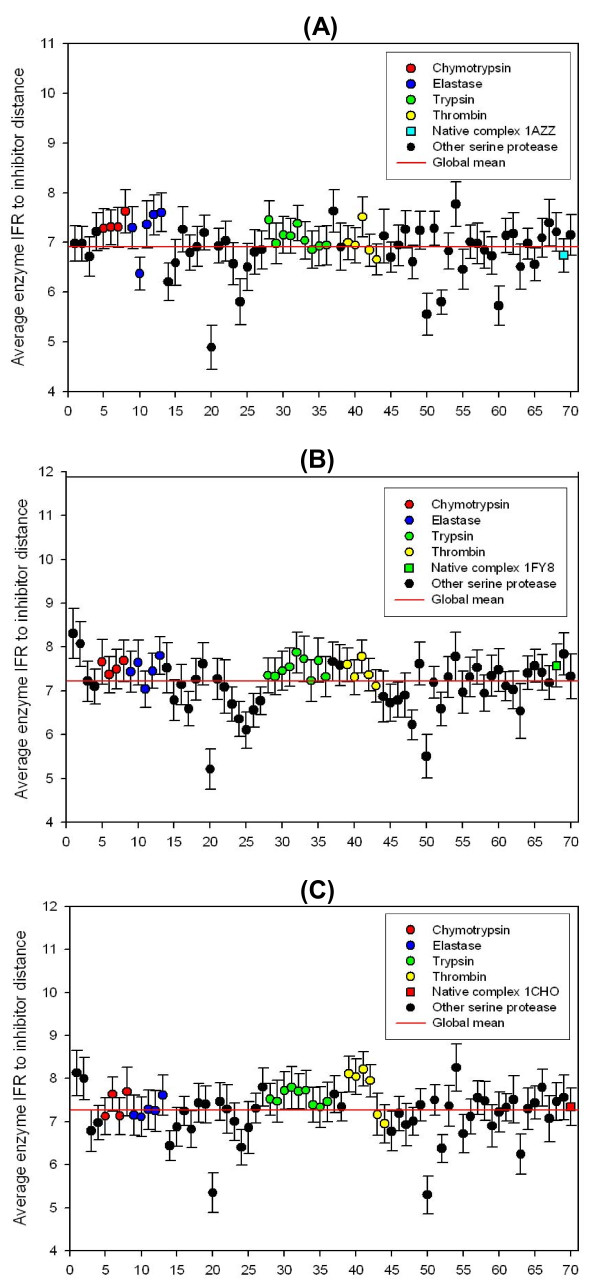
**Proximity of IFRs belonging to residues from 70 serine proteases to the closest IFR residues belonging to Ecotine (A), BPTI (B) and Ovomucoid third domain (C)**. Each point in the graph represents the mean and the standard error of the mean for the smaller distances encountered for each IFR residue of the enzyme to the closest IFR residue of the inhibitor. The horizontal line is representing the global average value for the measured distances.

We calculated the distances between enzyme and inhibitor residues at the interface for BPTI (Figure 6A), ecotine (Figure 6B) and ovomucoid third domain complexes with serine proteases (Figure 6C). All 70 serine proteases are presented using the same numbering (x-axis) as in Table 1, and the same subfamily grouping (colored symbols follow the colors of subfamilies as in Figures 2, 3 and 4). The y-axis shows the distances, measured in Ångstroms, with bars indicating a corresponding standard deviation for each point. The distances are the average values obtained by calculating the sum of the CA-CA (Alpha Carbons) distances for each amino acid belonging to the IFR ensemble from the enzyme side, to the closest amino acid from the IFR of the inhibitor, and then divided by the number of IFR residues at the enzyme site. A horizontal line represents the average global mean which was always near 7.0 Å for all three types of inhibitor complexes. In a prior paper [24], our research group demonstrated that 7.0 Å is the cut-off distance that optimizes the isolation, at the residue level, of first order of contacts (first coordination shell) in well-packed globular proteins. The observed 7.0 Å average global mean distance among CAs in enzyme-inhibitor complexes confirms that inhibitors and enzymes form well-packed complexes like those found in the core of globular proteins. Thus, it is plausible to conclude that complexes which present distances less than the average global mean contain spatial clashes. Those complexes can be further submitted to dynamics and minimization in order to rearrange these residues and diminish the steric clashes. We are set up to try to evaluate how many and which steric clashes would be removed by such procedure, however, this is yet to be done as a next step of our project.

## Discussion

The data collected, analyzed and presented in this work serves as a tool for understanding binding properties in general and protease specificity in particular, based on Interface Forming Residue (IFR) profile alignment.

The procedure we have developed for this work describes a): the ensemble of IFRs for any complex formed between a serine protease and an inhibitor and b): the differences in interface characteristics between various proteases with respect to specificity. From our data one may not extract a simple, concise and very straightforward rule for separation among proteases in terms of specificity (defined here mainly by IFR position occupancy and characteristics). Nature obviously prepared a mechanism for fine tuning of the activity that yields terms of specificity for each subfamily of serine proteases. There is obviously some space left for overlaps among IFR characteristics and positions that dictate the specificity for each sub family and consequently, what could have been a simple table with reduced complexity in terms of "colors" and characteristics of amino acids employed at each IFR position, became somewhat blurred. The data presented here are precise in pointing to such granular/spectral distribution in IFR occupancy that would be directly coupled to the specificity type of studied enzymes. It is our perception that the beauty of the adaptability of molecular mechanisms, expressed here in form of their specificity, can still be presented in a such simplified way (described in Figures 1 to 4) showing how specificity can be fine-tuned from different points of entry (multiple, rather than single): IFR positions and characteristics of amino acids that occupy those critical positions. The procedure we described may also open the possibility to yield some details that may account for differences in how strong might be the binding between an enzyme and an inhibitor (an issue we need to explore in the future with more details based on elaborated IFR tables and experimental data available in literature).

It has been established that increased structural plasticity in the binding pocket increases the variation of substrate size that can fit into the critical space directed toward the catalytic triad which correspondingly decreases the specificity [25]. Consequently, it is expected that the higher the stiffness around the binding pocket, the higher the selective pressure will be on a particular substrate. Specificity then can be seen as directly proportional to structural limitations imposed first by the size of the docking space and then by the physical and chemical characteristics of this space. By focusing our attention on the type of residues occupying the enzyme and inhibitor interfaces, we have set aside the evaluation of how plasticity of binding pocket influences enzyme specificity. This factor, however, is not taken as a non important one. In fact, we will be undertaking further examination in order to understand better how plasticity may be accounted for by IFR properties - therefore we will be tackling this task in the future. As a start, the residue types occupying the interface positions implicitly contain the sort of information which relates directly to plasticity of the site.

## Conclusions

The work presented here offers insight into the structure/function relationship of serine proteases. The superposition of structurally aligned backbones of serine proteases showed that they use essentially identical scaffolding and achieve a variety of specificities by varying surface residues. Previously, there have been attempts to modify the functionality of serine proteases. These attempts usually focus on several residues and generally result in the loss of enzyme activity. Our approach explains why and corroborates with the results presented by Ma et al. [26] and Novozymes Biotech, Inc. (Davis, CA, US) which patented a technology (Microbial trypsin mutants having chymotrypsin activity - United States Patent 20050037368) by confirming necessity for several IFR amino acids to be either substituted, or deleted ("no-show" event) or to be introduced in original protease sequence in order to transform trypsin to chymotrypsin.

Based on the results we are reporting here, we are poised to assemble, in the near future, the serine protease superfamily interface data resource as an expanding collection of sequence, structural, and functional information about the serine proteases interface forming residues. A combination of graphics, images and numerical data will be used to aid in the complete analysis of structure/function relationships that still require our attention before we may fully understand the precise role of each amino acid employed at the particular location by enzymes in order to achieve desired specificity.

## Methods

In order to provide a sufficient volume of data for analysis of the interfaces around active sites of serine proteases (even if there is no currently available information on complex formation of one particular protease with any specific substrate and/or inhibitor) we needed to employ the *in silico *approach for building corresponding complexes. The key feature of our work is mapping the IFR 3D profile into a 2D matrix--from a known enzyme-inhibitor structure to those with no known structure for such complex. Mapping is done after structurally aligning all serine proteases with non-redundant sequences.

### Selection of serine protease: structural data set

The PDB IDs for serine proteases were obtained from SCOP database [27] parseable files (release 1.73). All 1086 PDB IDs from family b.47 were selected. FASTA files relative to these IDs were obtained in the PDB. A BLAST search (blastp), using default parameters, was run to determine the percentage of identity between the selected sequences. Those sequences with lower than 95% identity were selected for further examination (meaning that there should be at least 12 to 15 different residues occupying corresponding positions among selected sequences. In general, the sequences are 240 to 260 amino acids long, which means that applied filtering eliminates structures obtained after single, or even after limited multiple, point mutations). From the resulting subset (which we named here as the "primary datamart"), further selection eliminated all but those structures showing the highest resolution (lower numerical values in Å) and best R-value. This yielded what we named as a "secondary datamart". The "secondary datamart" at this point contains 67 serine proteases. Those structures covered the following sub families: tryptase, chymase, chymotrypsins, elastase, exfoliative toxin, glutamyl endopeptidase, kallikrein, NS3 protease, streptogrisin, trypsin, venom, thrombin, serine proteases from prokaryotes (which we named as "miscellaneous-prokaryotes"), serine proteases from viruses (which we called "miscellaneous-virus") and serine proteases that do not fit any of the mentioned sub families which we called "miscellaneous". The above described division resulted in 15 "sub-families" on which we applied additional manual inspection (in order to re-confirm their classification) by consulting the following sources: PDBsum [28], BRENDA [29] and SCOP parseable files.

The list of all corresponding PDB IDs for serine proteases used in this work is presented in the Table 1.

### Selection of experimentally described enzyme/inhibitor structural complexes

From the 57 available PDB files containing inhibitor/serine protease complexes (data from January 2009), comprising 11 SCOP inhibitor families, the three largest families were selected and the same approach as described above was followed to select for what we named here as the three "prototype complexes". Complexes containing BPTI, ecotin and ovomucoid inhibitors correspond respectively to g.8, b.16 and g.68 SCOP subfamilies and are represented, respectively, by 1FY8 (inhibiting rat trypsin), 1AZZ (inhibiting crab collagenase) and 1CHO (inhibiting cattle alpha-chymotrypsin) PDB files. We named this group as "prototype complexes" because they represent the experimentally described complexes with structures deposited into the PDB.

### Structural alignment

Before the alignment procedure took place, we needed to edit the PDB files. The 1CHO.PDB file was edited to replace the three enzyme chain IDs (E, F, G) with a single "chain E". This was done in order to easily distinguish the enzyme chains from the inhibitor chain named "I". For the 1AZZ file, containing two chains, only chain A (enzyme) and chain C (inhibitor) were used (the PDB file contains a description of a hetero-dimer). The 1FY8 was not edited before further processing. In order to obtain an IFR ensemble for the serine proteases lacking an available structure for the complex with chosen inhibitor (or any inhibitor at all), we first structurally aligned all 70 selected serine proteases (67 from the "secondary datamart" plus 3 serine proteases from the "prototype complexes") using PrISM [30] and its set of default parameters, http://wiki.c2b2.columbia.edu/honiglab_public/index.php/Software:PrISM. The PrISM software also provided a multiple sequence alignment (being generated as an output obeying the structural alignment), which was saved and parsed to preserve both residue numbering and residue positions occupied in the structural alignment.

### Rigid body docking

**Figure 7:**
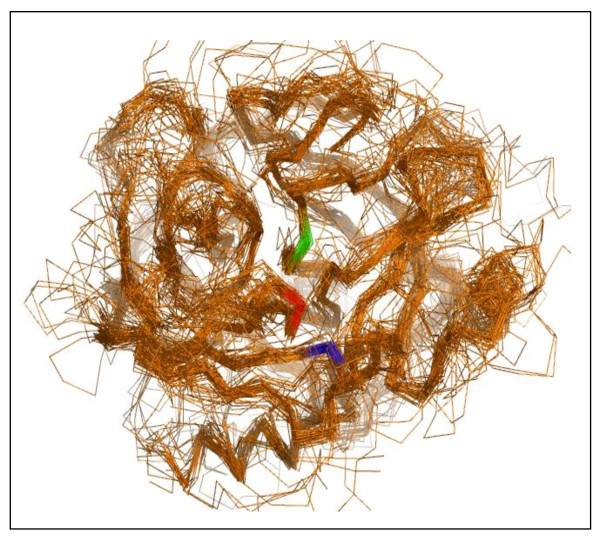
**Structural alignment of 70 different (sequence-wise non redundant) serine proteases aligned by PrISM package**. The image was produced using PyMOL. Only the main chain is represented. The following positions in chymotrypsinogen are highlighted: Ser 195 in green, His 57 in red and Asp 102 in blue.

**Figure 8:**
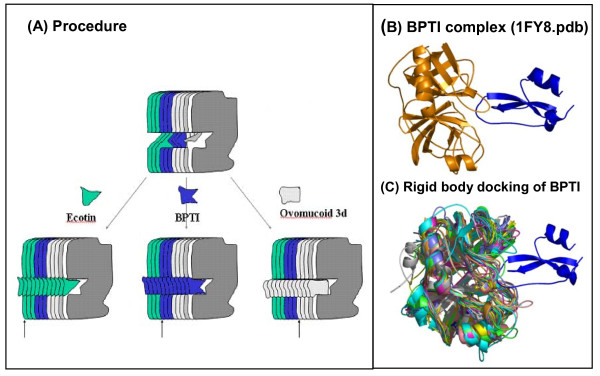
**Hard docking of inhibitor coordinates to selected serine proteases**. (a) Schematic diagram of the experiment for obtaining the structure of three different sets of 70 serine proteases with each one of the three selected inhibitors: Ecotine (green), BPTI (blue) and Ovomucoid third domain (light gray). The arrows placed below each of the three sets of 70 structurally aligned serine proteases are indicating that only one structure had experimental coordinates for both the serine protease and its corresponding inhibitor (indicated by the corresponding color). All the other proteases "received" that inhibitor in a position identical to the one found in the experimentally determined enzyme/inhibitor complex (one of three "prototype complexes"). From each obtained set, the IFR ensemble was extracted and analyzed; (b) structure of the BPTI/protease complex as found in 1FY8.pdb; (c) BPTI is "rigid body docked" into the complementary 70 structurally aligned serine proteases. One of those 70 enzyme structures is identical to the one presented in (b).

**Figure 9:**
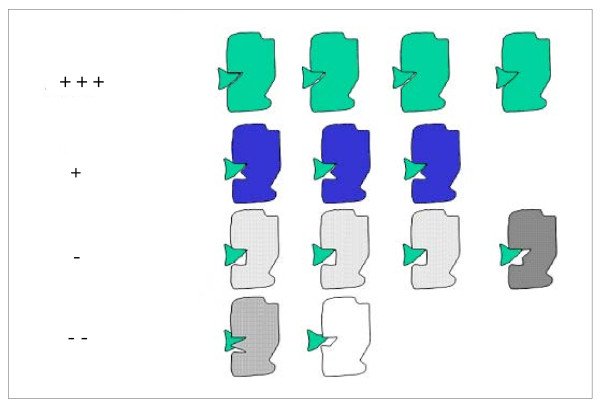
**Qualitative evaluation of the space fit between inhibitor and serine proteases**. The first set of complexes obtained as described in Figure. 6 above was de-convoluted in order to schematically demonstrate space compatibility of the BPTI inhibitor and the binding pocket of the complementary 70 different proteases. The "+"and "-" signs were manually introduced to qualitatively "quantify" visual complementarity of the surfaces of the inhibitor and corresponding binding pocket. We used those structures to exclusively identify the IFRs, not to evaluate the binding compatibility.

The term "rigid body docking" is used here to describe what was done in order to get complexes of each of the 67 serine protease structures bound to 3 different inhibitors. Effectively what we did was added the inhibitor coordinates to each PDB file from the "secondary datamart". In order to do so, we first needed to guarantee that the inhibitor molecule would be added to any of the enzyme molecules in exactly the same fashion as it did in the "prototype complex". This guarantee was provided by aligning a pair of structures where the first was the enzyme structure from the original "prototype complex" (e.g. 1FY8) and the second was from the PrISM output file - one of the 70 structurally aligned enzymes (e.g. 1FY8_PrISM). This additional structural alignment was performed with PyMOL [31] software, using the command line "align (1FY8, 1FY8_PrISM)", which maintained the PrISM generated coordinates of the 1FY8_PrISM structure and superimposed the enzyme coordinates from a selected "prototype complex". Consequently, this procedure also relocated the inhibitor coordinates from the "prototype complex". The newly obtained coordinates for the inhibitor were then extracted from the PyMOL output (PDB formatted) file and used to edit the PrISM output file for the other 69 serine proteases. By doing so, we included the inhibitor coordinates now fitting to a selected enzyme in a position exactly equivalent to the one that the inhibitor assumed in the "prototype complex". The three sets (each containing 70 complexes) were therefore formed. The details of the procedure described above are illustrated in Figures 7, 8 and 9. Upon completing the procedure, all structures containing a complex between a serine protease (one of 70 of them) and an inhibitor (one of the three selected ones) could be described as spatially oriented in a consistent and unique manner (obeying structural alignment) and having the inhibitor in a position consistent with the one observed and described experimentally.

As the primary purpose was to delineate a general area defined as an interface, additional precision, as obtained by minimization and dynamics procedures (generally applied to remove space-clashes), was not considered critical and therefore not applied in this stage of the work. It is important to note that many complexes are in fact "forced" to be formed *in silico *by a "rigid body docking" procedure, but due to various factors, including insufficient shape complementary between the inhibitor side and corresponding binding pocket on the enzyme side, they would form with a certain degree of instability and would therefore be short lived. A qualitative description of this scenario is described in Figure 9 (also see Figure 6. for an estimate of the degree of space clashing among enzymes and inhibitors in all *in silico *formed complexes).

### Surface determination in rigid body docked complexes

The three sets of 70 enzyme-inhibitor complex structures were also studied with respect to solvent accessibility of surface amino acids (calculated both before and upon inhibitor binding). The change in solvent accessibility of surface amino acids for both inhibitor and enzyme (in isolation and upon binding) was calculated by the Surfv algorithm [18]. Residues with a change in solvent accessibility were compiled into the ensemble of IFRs.

### Interface Forming Residues Table

For each set of 70 complexes, we mapped all of the sequence positions that belonged to the IFR ensemble (table shown at Figure 1). We took the native trypsin-BPTI complex (1FY8) as a reference for sequence numbering because it follows standard serine protease sequence numbering. All residues showing a detectable loss of accessible area were labeled in the multiple structure-based sequence alignment obtained with PrISM according to the residue positions in the sequence of 1FY8 chain E. In the case of gap in the reference sequence, a letter was added to the previous number (designating the position of the particular amino acid in a given sequence), so that the label keeps following the reference sequence numbering. Columns without at least one residue labeled as an IFR were eliminated to facilitate the analysis. For the case of BPTI, 36 residues occupying IFR positions were compiled (table shown at Figure 2) and ranged from positions 37 to 228 (also see Figure 1). Corresponding numbers for the ecotine and ovomucoid third domain are 40 IFRs from 37 to 226 and 34 IFRs from 37 to 217A, respectively. The multiple alignment of IFRs for the 70 serine proteases complexed with BPTI is presented in the Figure 1 (the corresponding tabular data for ecotine and ovomucoid inhibitor are not shown here). The derivatives from the tables with 70 serine proteases bound to BPTI, ecotine, and ovomucoid third domain are presented in the tables shown at Figures 2, 3 and 4; there we have only those columns which had some prevalent residues occupying that specific position. Particular differences among 4 principal classes of serine proteases were aimed. In other words, some positions were eliminated as they had a same residue present in each of the four sub-families of serine proteases (such as the position of HIS 57 which does not contribute to differentiation among 4 subfamilies). Those positions which were not identified as an IFR for the particular pair of serine protease and inhibitor are presented by a "-" sign. It is important to mention that the Asp_102 is not seen in Figure 1 due to a very peculiar characteristic of the catalytic pocket (as explained previously in the "Background" session).

Once we constructed the 3 sets of 70 complexes of proteases with respective inhibitors, we made an inquiry into the relationships between structural characteristics and specificity of enzymes. We calculated the difference in residue type occupancy of the total enzyme surface and the respective interfaces. The goal was to find if the interface was different from the rest of the protein surface in terms of types of residues present. Using the Surfv algorithm, we calculated the total enzyme surface area and determined the fraction occupied by each of four classes of amino acids. The same procedure was applied for the IFR area. Amino acid classes are as indicated: Charged, Polar, Hydrophobic and Glycine (a single member sub group of polar). The results of this inquiry are shown in Figure 5, as well as tabulated in the Table 2, and are discussed in more detail in results and discussion sections.

### Amino acid classification and color coding

Before we can discuss our findings, it is necessary to explain the founding principles for amino acid classification (and their color coding) that we adopted for this work. In this paper we decided to pay special attention to three amino acid classes (hydrophobic, polar and charged) and also to treat, in a separate way, the three amino acids **G**, **C **and **H**. In the case of **Gly**, its occasionally given separate consideration because of its peculiarity: **Gly **has one hydrogen atom instead of the side chain (in a sense, Gly residues received an individual treatment here just as in the Ramachandran plot [32], for example). In case of the **Cys**, special treatment is given because it is the only residue that forms disulfide bridges. Lastly, **His is treated separately **because this residue is "neutral polar" at physiological pH but positively charged when protonated.

The amino acid color code is an adaptation of "aminochromography" suggested by William Taylor [33]. Residues **AVLIMFP **are colored grey (small hydrophobic [**A **and **P]) **and also large hydrophobic [**VLIMF**]). Residues **STYNQWG **are colored green (polar - we are including **Y **and **W **to this ensemble although both residues have aromatic rings: phenol and indole, respectively). Those aromatic rings are often considered as another important feature for Y and W - the source of their hydrophobicity. The hydrophobic character of **W **and **Y **is often imposed above their polar characteristics. However, we consider **W **as an ambivalent residue (polar and hydrophobic) and **Y **as a polar residue. Residues **D **and **E **are colored red (negatively charged). Residues **R **and **K **are colored blue (positively charged). Residue **C **is colored yellow (disulphide bridge forming), and residue **H **[positively charged but neutral at neutral pH] is coloured cyan (light-blue).

The classification of amino acids follows the scheme given in "Structural Bioinformatics", page 18 [34] and is supported by hydrophobicity scales by Janin [35], Kyte and Doolitle [36] and Rose [37]. It is known that some of the hydrophobicity tables cited here (and in other papers found in general literature) present an apparent contradiction with respect to classification of certain amino acids. For example G and W are often a subject of controversy in terms of being considered polar or hydrophobic. We needed to decide which option to use in our paper, and the preceding description defines which one we opted for. It is clear therefore that an alternative classification is also possible although, we would consider it less precise.

### Validation of Methodology

To validate our methodology for defining IFRs through "hard docking", we selected 16 native complexes (not present in the "secondary datamart") of serine proteases bound to BPTI--the inhibitor with the largest number of native complexes available in the PDB (Table 3 lists the PDB IDs of the selected complexes). We investigated how successful our methodology was at predicting IFR residues. Namely, by using the 1FY8 complex as a reference and transferring the inhibitor coordinates to the enzyme chains of the sixteen complexes, we compared real IFRs and ones obtained by rigid body docking. This was done by aligning the enzyme chains of the sixteen complexes using PyMOL and rigid body docking the inhibitor of 1FY8. The docking results were then compared to those reported for native bound inhibitors. Afterwards, we used the Surfv algorithm to determine the residues composing the interface (IFR) of the sixteen *in silico complexes*. The same algorithm was used to determine the IFRs in the respective native complexes. A comparison between rigid body docking and native IFR in natural complexes was then possible. From these results, a "confusion matrix" [38] was generated. Detailed analysis of corresponding data is given in results and discussion sections.

## Authors' contributions

CR: DB and datamarts preparation, structural alignment, PyMOL alignment, IFR tables, figures, manuscript. RT: DB preparation, structural alignment, IFR tables, figures, manuscript, PrISM maintenance. IM: DB maintenance. AM: DB maintenance. RMM: Experiment validation. CH: Experimental validation. IAPN: calculating values for the tables and figures of frequency of occurrence for surface and interface residues. JGJ: project coordination. MS: project coordination and oriented this work. GN: project supervisor, elaboration of techniques for hard docking and IFR assemblies. All authors read, revised and approved the final manuscript.
